# Enhancing Ocular Surface in Dry Eye Disease Patients: A Clinical Evaluation of a Topical Formulation Containing Sesquiterpene Lactone Helenalin

**DOI:** 10.3390/ph17020175

**Published:** 2024-01-30

**Authors:** Dalia Ng, Juan Carlos Altamirano-Vallejo, Jose Navarro-Partida, Oscar Eduardo Sanchez-Aguilar, Andres Inzunza, Jorge Eugenio Valdez-Garcia, Alejandro Gonzalez-de-la-Rosa, Andres Bustamante-Arias, Juan Armendariz-Borunda, Arturo Santos

**Affiliations:** 1 Tecnologico de Monterrey, Escuela de Medicina y Ciencias de la Salud, Monterrey 64849, Nuevo Leon, Mexico; dalia.ngaleman@gmail.com (D.N.); jcaltamirano@e-retina.com (J.C.A.-V.); josenavarro@tec.mx (J.N.-P.); a01206795@tec.mx (O.E.S.-A.); dr.andresinzunza@gmail.com (A.I.); jorge.valdez@tec.mx (J.E.V.-G.); agonzalez1@tec.mx (A.G.-d.-l.-R.); armdbo@gmail.com (J.A.-B.); 2 Grupo Oftalmologico Acosta, Hospital Puerta de Hierro, Zapopan 45116, Jalisco, Mexico; 3 Centro de Retina Medica y Quirurgica, Hospital Puerta de Hierro, Zapopan 45116, Jalisco, Mexico; 4 Clinica de Oftalmologia Sandiego, Medellin 050012, Colombia; abustamante.eye@gmail.com; 5 Centro Universitario Ciencias de la Salud, Universidad de Guadalajara, Guadalajara 44340, Jalisco, Mexico

**Keywords:** ocular tolerability, *Arnica*, MMP-9, conjunctival impression cytology, meibomiography

## Abstract

The aim of this work was to assess the tolerability, safety, and efficacy of an ophthalmic topical formulation containing helenalin from *Arnica montana* and hyaluronic acid 0.4% (HA) in patients with mild-to-moderate Dry Eye Disease (DED) exhibiting positive Matrix Metalloproteinase 9 (MMP-9) test results. Tolerability and safety were evaluated in 24 healthy subjects. Participants were instructed to apply one drop of the formulation three times a day in the study eye, for 2 weeks, followed by a clinical follow-up of 21 days. Efficacy was studied in 48 DED patients randomized into Study (Group 1/receiving the studied formulation) or Control (Group 2/Receiving HA 0.4% eye lubricant) groups for 1 month. Assessments included an MMP-9 positivity test, conjunctival impression cytology (CIC), Ocular Surface Disease Index (OSDI), non-invasive film tear breakup time (NIBUT), non-invasive average breakup time (NIAvg-BUT), ocular surface staining, Schirmer’s test, and meibomiography. A crossover design with an additional 1-month follow-up was applied to both groups. Healthy subjects receiving the studied formulation exhibited good tolerability and no adverse events. Regarding the efficacy study, Group 1 exhibited a statistically significant reduction in the MMP-9 positivity rate compared to Group 2 (*p* < 0.001). Both Group 1 and Group 2 exhibited substantial improvements in OSDI and NIBUT scores (*p* < 0.001). However, Group 1 demonstrated a significant improvement in NI-Avg-BUT and Schirmer’s test scores (*p* < 0.001), whereas Group 2 did not (*p* > 0.05). Finally, after the crossover, the proportion of MMP-9-positive subjects in Group 1 increased from 25% to 91.6%, while Group 2 showed a significant decrease from 87.5% to 20.8%. Overall, the topical formulation containing sesquiterpene helenalin from *Arnica montana* and hyaluronic acid was well tolerated and exhibited a favorable safety profile. Our formulation reduces DED symptomatology and modulates the ocular surface inflammatory process; this is evidenced by the enhancement of CIC, the improvement of DED-related tear film status, and the reduction of the MMP-9 positivity rate.

## 1. Introduction

Nature has long been recognized as a valuable repository of compounds possessing unique biological activities relevant to human health [1,2]. Among the vast array of secondary metabolites found in plants, sesquiterpene lactones (SL) have garnered significant attention for their capability to modulate inflammation pathways induced by oxidative stress [3,4,5,6,7]. SLs are isolated from *Asteraceae* species and consist of more than 8000 compounds, with each offering a variety of modifications and structures [4,5,6,7,8,9]. They have a structural foundation of 15 carbons and a fused α-methylene-γ-lactone ring. SL compounds exhibit a broad spectrum of potential health benefits, encompassing anti-inflammatory [8], antitumoral [3,6], antioxidant [9], neuroprotective [10], hepatoprotective [11], immune-stimulating [4,7], antimicrobial [12], and antiparasitic properties [13]. Notably, helenalin, a pseudoguaianoloide sesquiterpene lactone derived from *Arnica montana* and *Arnica chamissonis* ssp. *foliosa*, has recently regained attention as a lead compound for inflammation treatment [14,15,16,17,18]. Its anti-inflammatory mechanism diverges from that of non-steroidal anti-inflammatory drugs (NSAIDs), attributed mainly to its potent inhibition of nuclear factor kappa-light-chain-enhancer of activated B cells (NF-κB) signaling [6,15]. Furthermore, helenalin-exposed T-Helper Cluster of Differentiation 4+ (TCD4+) cells have shown reduced interleukin-2 (IL-2) production and CD25 expression, suggesting helenalin’s potential as an anti-inflammatory therapy [18,19,20]. This is particularly significant in chronic inflammation diseases, where conventional treatments carry risks of adverse events and non-adherence [7,8,21,22,23,24]

Dry Eye Disease (DED) is commonly defined as a “multifactorial disease of the tears and ocular surface that results in symptoms of discomfort, visual disturbance, and tear film instability with potential damage to the ocular surface. It is accompanied by increased osmolarity of the tear film and inflammation of the ocular surface” [25]. DED can be diagnosed by the Tear Film Ocular Surface Dry Eye Workshop II (TFOS DEWS II) criteria, based on a positive symptom score with the Dry Eye Questionnaire (DEQ-5) and Ocular Surface Disease Index (OSDI), and one of the following homeostasis markers: non-invasive tear breakup time of <10 s (Oculus Keratograph 5M); the highest osmolarity value of ≥308 mOsm/L among eyes or an interocular osmolarity difference of >8 mOsm/L (TearLab Osmolarity System); or >5 corneal spots, >9 conjunctival spots or lower/upper lid-wiper-epitheliopathy staining of ≥2 mm length and ≥25% width (Oculus Keratograph 5 M) [26].

DED is a highly prevalent condition. The prevalence of DED varies widely between studies. In a cross-sectional study conducted in the United Kingdom, the prevalence of DED in healthy subjects was found to be 32.1% [26]. In an American study, the prevalence of DED, based on weighted estimates, was projected to be 6.8% of the US adult population, equating to approximately 16.4 million people. It was estimated that more than 16 million US adults have been diagnosed with DED [27]. The estimated pooled prevalence of DED in Asia was 20.1% (95% CI of 13.9–28.3%) [28]. The prevalence of DED in the Mexican population was 41.1% (95% CI of 38.6–43.6%) [29].

As was established previously, DED is characterized by ocular surface inflammation due to hyperosmolarity and is associated with excessive reactive oxygen species production, oxidative stress, and lymphocyte infiltration. Patients with DED experience a marked T-cell infiltration of the cornea and conjunctiva accompanied by an increase in pro-inflammatory cytokines and Matrix Metalloproteinase secretion (MMPs) (including MMP-9) [30,31,32]. Importantly, pro-inflammatory interleukins (IL-1, IL-6, IL-8) and tumor necrosis factor TNF-α further contribute to chronic ocular surface damage [30,31,33]. In this context, NF-κB, a protein complex that controls the transcription of multiple pro-inflammatory cytokines in response to TNF-α signaling, emerges as a key regulator of ocular surface inflammation, responding to diverse stimuli involved in DED [30,31,33,34].

Currently, lubricant eye drops represent the standard approach for DED management [33]. However, due to the inflammatory component of DED, some anti-inflammatory agents, such as topical cyclosporine, corticosteroids, and NSAIDs, are frequently included in DED therapy in selected patients [33]. Despite their efficacy, these treatments are associated with potential adverse effects such as ocular pain, irritation, cataract formation, and ocular hypertension, therefore limiting their long-term application [31,32,33,34,35]. Furthermore, adherence to DED treatments such as cyclosporine is significantly low [35]. In many cases, this is related to the treatments’ insufficient alleviation of inflammation-related symptoms, with some studies reporting compliance rates as low as 10.2% [30,36]. Consequently, a definitive curative anti-inflammatory therapy for DED remains elusive [33,34].

This phase I/II study aimed to investigate the safety and tolerability of an ophthalmic formulation containing helenalin, a blocking agent of NF-κB signaling, extracted from *Arnica montana* L. and hyaluronic acid in the eyes of healthy subjects, as well as its clinical efficacy in the eyes of patients with mild-to-moderate DED.

## 2. Results

### 2.1. The Studied Formulation Is Tolerable and Safe in Healthy Volunteers

Twenty-four female and male healthy volunteers were included in the tolerability and safety study group. The mean age was 36.4 ± 9.82. Thirteen subjects (54.16%) were men and eleven (45.83%) were female, with twelve right eyes (50%) and twelve left eyes (50%). After enrollment, participants were instructed to regularly apply one drop of the formulation three times a day to the study eye for 21 days. Concerning safety and tolerability outcomes, no serious adverse events (SAEs) were associated with the administration of the ophthalmic studied formulation during the 21-day follow-up period. Table 1 presents a comprehensive overview of all recorded AEs, each of which exhibited a transitory nature following drop application and spontaneously resolved within a brief timeframe (<10 min).

**Figure 1:**
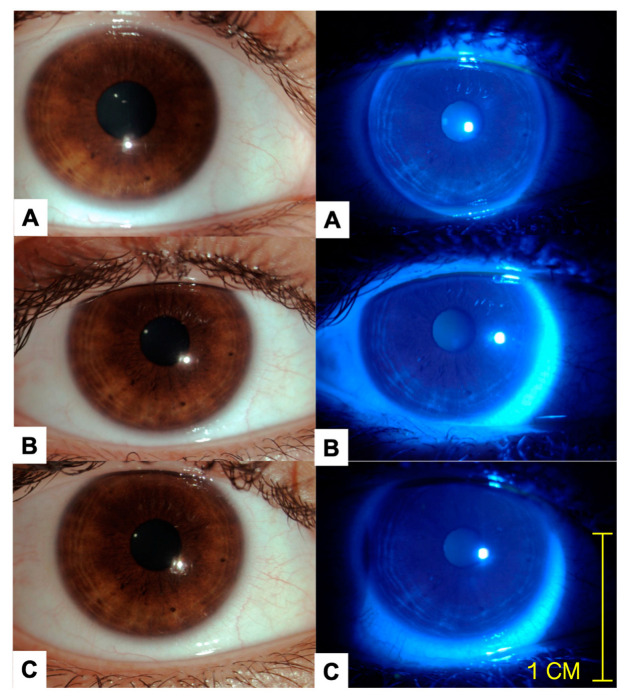
Representative images of the same patient on days 1 (**A**), 14 (**B**), and 21 (**C**) are provided. The images in the left column are color photos acquired during slit lamp examination under white light, while the images in the right column are color photos acquired with ocular surface staining using a cobalt blue filter and 1.0 mg fluorescein sodium ophthalmic strips (FluoroTouch^®^). This procedure was performed at every visit. No corneal or conjunctival staining was detected after 21 days of clinical follow-up with daily use of the studied formulation. There was no evidence of inflammation, swelling, or discharge following the instillation of the ophthalmic formulation at the day 21 clinical visit.

Furthermore, the clinical evaluation revealed no noteworthy ocular abnormalities or significant alterations. None of the 24 subjects exhibited significant changes in BCVA, IOP, or cECD throughout the clinical follow-up. Registered values for the previously mentioned variables are presented in Table 2. Representative images of the ocular surface at baseline and the final visit are shown in Figure 1.

### 2.2. The Ophthalmic Studied Formulation Ameliorates DED Symptoms, Improves Tear Film Objective Evaluations, Increases the Number of Negative MMP-9 Tests, and Improves CIC in Patients with Mild-to-Moderate DED

**Figure 2:**
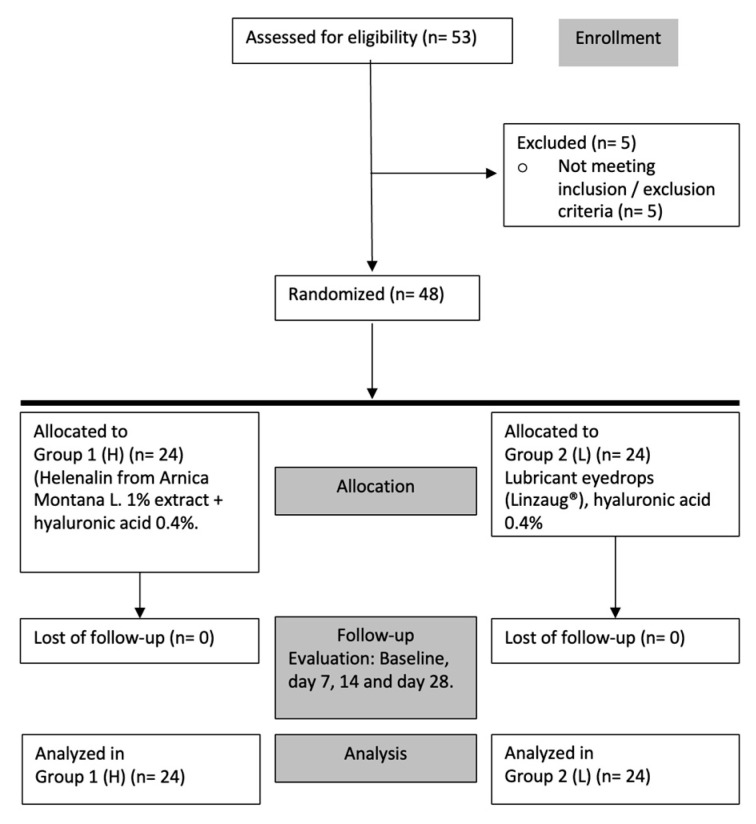
Efficacy evaluation study. Flow chart for enrollment, allocation, evaluation, and analysis.

To explore the effect of the studied formulation containing helenalin on the ocular surface health of DED patients, a phase II clinical study to evaluate the efficacy of the ophthalmic formulation was performed. The efficacy study included patients with mild-to-moderate DED meeting all inclusion criteria. Data analysis for 48 subjects, 24 for the Study group (Group 1) and 24 for the Control group (Group 2), completed the follow-up period (Figure 2).

The demographic and clinical characteristics of the subjects and the eyes involved in the efficacy assessment study are shown in Table 3. The baseline characteristics of subjects and eyes from Group 1 (studied formulation) and Group 2 (Control) were similar. There was no statistical significance between groups.

**Figure 3:**
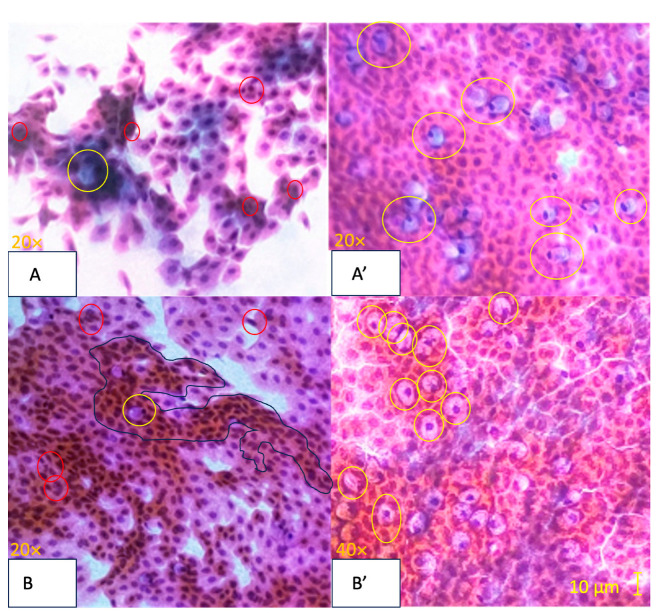
Representative images of Nelson-graded conjunctival impression cytology specimens stained with Papanicolaou stain [37]. Image (**A**) (Baseline): Stage 0, Nelson Grade 3. Abundant cellularity with 80–100 goblet cells/mm^2^ (marked by yellow circles). Moderate intercellular separations with a modified cytoplasmicto-nucleus ratio favoring cytoplasm. Mild inflammation and a slight increase in apoptosis (marked by red circles). Image (**A’**) (1-Month): Stage 0, Nelson Grade 0. Abundant cellularity is evident with more than 400 goblet cells/mm^2^ (marked by yellow circles). The cells are cohesive. There is a balanced cytoplasmic-to-nuclear ratio. No inflammatory cells or apparent apoptosis are observed. Image (**B**) (Baseline): Stage 0, Nelson Grade 2. Abundant cellularity with goblet cells ranging from 200 to 300 cells/mm^2^ (marked by yellow circles). Frequent slight intercellular separations with a modified cytoplasmic–nucleus relationship favoring cytoplasmic features. Mild inflammation and metaplasia (surrounded by a blue perimeter) are noted, along with a slight increase in apoptosis (marked by red circles). Image (**B’)** (1-Month): Stage 0, Nelson Grade 1. Abundant cellularity with goblet cells ranging from 300 to 400 cells/mm^2^ (marked by yellow circles). The cells are cohesive or with discrete intercellular separations. There is a balanced cytoplasmic-to-nuclear ratio. Minimal inflammation and minimal apoptosis are observed.

**Figure 4:**
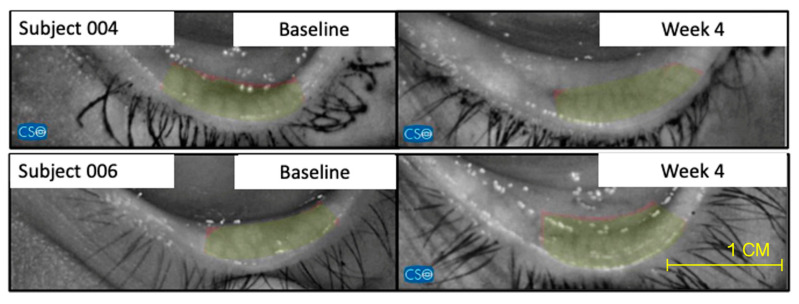
Representative images of 2 patients of the studied formulation group (Group 1) of meibomiography, taken with the Schwind Sirius^®^, that show notable improvement between baseline and week 4. This significative improvement was not observed in the Control group (Group 2).

Regarding efficacy, there was a statistically significant difference between baseline and 1-month for the qualitative evaluation (OSDI) results in the studied formulation group (Group 1) and the commercially available lubricant eye drop (Group 2). Specifically, the results depicted a change of 21.31 ± 3.21 vs. 10.58 ± 4.21 in Group 1 and 21.74 ± 15 vs. 11.23 ± 8.95 in Group 2. There was also a statistically significant difference for the quantitative evaluation NIBUT when comparing baseline and 1-month observations in the studied formulation group (Group 1) (8.39 ± 5.86 vs. 14.53 ± 4.53) and Group 2 (8.43 ± 4.82 vs. 13.83 ± 5.69). Only the Study group (Group 1) showed a statistically significant difference in the NIAvg-BUT measurement when comparing baseline and 1-month observations (10.46 ± 4.19 vs. 14.24 ± 3.66), whereas Group 2 did not (9.85 ± 4.13 vs. 11.73 ± 5.82). The studied formulation group also exhibited a statistically significant difference in Schirmer’s test measurements (15.83 ± 5.4 vs. 20.05 ± 4.37), whereas the Control did not (16.11 ± 5.1 vs. 17.83 ± 6.28). Concerning MMP-9, Group 1 showed a clear positive change (statistically significant difference) between baseline and 1-month visit, since only 6 of 24 subjects presented positive MMP-9 tests by the end of the follow-up at 1-month (100% vs. 25%). This difference was not observed in the two groups (24/24 100% vs. 21/24 87.5%). Additionally, after 1 month of treatment with the studied formulation, there was a remarkable improvement in the morphology of the ocular surface, as all subjects showed normal impression cytology at the end of the follow-up (8/24 33.3% vs. 0/24 0.0%). The commercially available lubricant eye drops group (Group 2) did not show a statistically significant difference in the CIC (7/24 29.1% for both baseline and 1-month). These results are presented in Table 4 and Table 5. CIC representative images are shown in Figure 3. In reference to the Ocular Surface Staining score (OSS) with fluorescein (F) and lissamine green (LG) dye, the studied formulation group showed a statistical difference between the baseline vs. 1-month visit (*p* = 0.0367). Group 2 did not show a statistically significant difference (Table 6). Finally, concerning meibomiography by Schwind Sirius^®^, all subjects of the Study group showed a relevant improvement between baseline and 1-month clinical visits (Figure 4).

After a crossover without a washout period, there was a supplementary 1-month follow-up period. A noteworthy transformation was evident among subjects in Group 1 (without DED treatment) who tested positive for MMP-9 (6/24 25%), as their numbers surged to 22/24 (91.6%) after the crossover. Conversely, for participants in Group 2, this trend reversed after transitioning to the helenalin-based topical formulation, with the positive MMP-9 cases decreasing from 18/24 (87.5%) to 5/24 (20.8%) after the crossover.

## 3. Discussion

In our safety and tolerability study, we did not observe ocular surface irritation signs such as hyperemia, conjunctival or corneal epithelium punctate keratitis, or ocular surface staining. No SAEs were associated with the administration of the studied formulation during the 21-day follow-up period of the phase I clinical study or during the 1-month clinical follow-up of the phase II study. Consequently, our studied formulation was considered safe and non-irritating according to the Pharmacopeia of Mexico [38,39]. These results support the safety and tolerability of our studied formulation [40].

In our phase II clinical study, our findings demonstrated that the studied formulation containing helenalin from *Arnica montana* L. and hyaluronic acid 0.4% (Group 1) improved the results of the OSDI test in patients with mild-to-moderate DED. Additionally, Group 1 showed statistically significant differences in quantitative anatomical evaluations. The baseline-versus-1-month evaluation of Group 1, treated with the formulation containing helenalin, showed a significant difference in NI-BUT and NIAvg-BUT scores. Significant differences in Schirmer’s test measurements were also observed. All subjects in Group 1 showed an improvement in meibomiography using the Schwind Sirius^®^ device. Furthermore, no serious adverse events were associated with the administration of the studied formulation during the 1-month clinical follow-up of the phase II study.

It is important to emphasize that there was a statistically significant difference between the baseline and 1-month positivity rate of MMP-9, in Group 1. After the use of a formulation containing helenalin, only 25% of patients in Group 1 tested positive for MMP-9 at the end of the follow-up compared to 100% at the baseline visit. There was also a statistically significant difference in Group 1′s CIC between baseline and 1-month. All eyes with morphological abnormalities of the conjunctival mucus and epithelium showed normal CIC results at the end of the follow-up period in the Study group (*p* = 0.0078), resulting in a decrease in squamous metaplasia of ≥2 grades in 62.5% of the subjects and at least >1-grade improvement in 100% of subjects. All eyes in Group 1 showed significant improvement in the number, density, and size of goblet cells.

Contrastingly, Group 2 did not show a statistically significant difference in NIAvg-BUT and Schirmer’s test measurements after the 1-month treatment period with the control solution. Group 2 did not exhibit a statistically significant difference in the MMP-9 positivity rate or CIC. Furthermore, none of the eyes in this group showed improvement in ocular surface morphology.

Interestingly, after the crossover, the number of subjects with positive MMP-9 levels in Group 1 significantly increased from 25% to 91.6%. On the other hand, subjects in Group 2 decreased from 87.5% to 20.8% at the end of the crossover. The increase in the positivity rate of MMP-9 in Group 1 after switching to the control formulation, as well as the decrease in the positivity rate in Group 2 after switching to the studied formulation, provides mechanistic evidence of the time-dependent anti-inflammatory effects of topical helenalin on the ocular surface. This improvement may be attributed to the anti-inflammatory effects of helenalin. A reduction in inflammation may contribute to the enhancement of Meibomian gland function [41].

Although the improvement in most of the goals of DED is notable at 1-month, it is not exceptional or unprecedented, according to previous reports on other active ingredients. Nutraceuticals and various drugs have been reported to produce statistically significant changes in the treatment objectives for DED within a 4–6-week timeframe [42,43,44]. For instance, certain studies utilizing oral Omega-3 and berry extracts have shown a statistically significant reduction in tear evaporation rate, a marked improvement in dry eye symptoms, a notable increase in tear secretion, and significant alleviation of subjective eye fatigue symptoms [42,43,44]. Additionally, other topical ophthalmic drugs have demonstrated noticeable improvements in tear film status within 2–4 weeks [45,46]. Perfluorohexyloctane ophthalmic drops have shown statistically significant improvements in the Eye Dryness Score as early as week 2, as well as significant changes in total corneal fluorescein staining at week 2 [46]. Furthermore, the ONSET-1 trial (a phase IIb clinical trial of Varenicline for DED treatment) demonstrated significant improvement in the primary outcome (Schirmer’s test score) at 28 days and improvement in the Eye Dryness Score [45]. Therefore, the rapid activity of our studied formulation is comparable with other topical or oral drugs.

In terms of the rationale for the frequency of treatment, there is no consensus or definitive evidence establishing the superiority of one dosing frequency over another in treating DED with HA [47]. Hynnekleiv et al., in their recent review of the literature on HA for DED, noted: “Drop frequency in treatment studies varied from 2 to 8 drops per day.” Improvements in therapeutic goals have been observed even with a drop frequency lower than three times per day [48,49]. Hynnekleiv et al. also remarked: “There was no clear pathophysiological or evidence-based rationale for the selected drop frequencies in these studies. Furthermore, none of the studies were designed to identify the optimal drop frequency for HA treatment”. They concluded by identifying two significant gaps in the literature: 1. the absence of studies investigating the ideal drop frequency for HA-containing eye drops, and 2. a lack of sufficient evidence to favor any specific HA formulation over others [47]. It is important to note that while topical HA was effective in improving clinical outcomes in our assay, the studied formulation in our research demonstrated a greater magnitude of effect and more pronounced clinical improvement.

Several studies have found that conjunctival chronic inflammation and goblet cell loss are correlated with the clinical severity and level of ocular surface inflammation in aqueous tear deficiency [50,51]. Recently, it has been described that an uncontrolled increase in MMP-9 levels and activity has been detected in the tear film of patients with DED, together with goblet cell loss and chronic inflammation [52,53,54,55,56]. The goblet cell loss and inflammation has been evaluated clinically by CIC in DED patients, whereas MMP-9 activity has been tested clinically by InflammaDry^®^.

Previously, Aragona et al. described CIC changes in eyes treated with sodium hyaluronate for 3 months, concluding that the long-term wound healing properties of sodium hyaluronate are beneficial for the treatment of DED, resulting in a significant improvement in CIC [57]. Recently, Buzzonetti and colleagues reported the safety and effectiveness of a combination of HA 0.2% and arnica extract 0.1% in reducing DED-related symptoms in pediatric patients with DED and allergic conjunctivitis. These studies agree with our findings using the studied formulation containing helenalin from *Arnica montana* combined with HA. It is possible that using helenalin extracts in ophthalmic formulations instead of *Arnica montana* itself could be related to a better efficacy profile. This is based on our observation of significant changes in ocular surface characteristics and MMP expression with the studied formulation. However, it is important to note that this statement requires validation through a new trial.

On the other hand, Ryu and colleagues also found that after short-term treatment for 1 month with corticosteroids, the use of topical steroids showed a greater improvement in DED symptoms in MMP-9-positive patients compared to MMP-9-negative subjects [58]. These results with topical corticosteroids are similar to our findings, where improvement in DED is observed in patients treated by the studied formulation containing helenalin. This could be explained on the basis of the anti-inflammatory effect of both strategies. However, randomized clinical trials of steroids in the context of DED have produced inconsistent outcomes in terms of the efficacy of steroids [59,60,61].

We believe that the clinical efficacy of our studied formulation lies in its ability to regulate the chronic inflammation of the ocular surface (CIOS) process. CIOS is a key contributing factor to DED-related symptoms and cellular damage [30,62,63]. It is well known that CIOS stimulates the expression of pro-inflammatory cytokines, chemokines, and MMPs through NF-κB pathway activation [55,56,57,58].

The exact mechanisms behind NF-κB activation in DED are not fully understood. Still, oxidative stress and continuous tear film hyperosmotic stress are believed to contribute to its dysregulation, leading to persistent inflammation in the cornea and conjunctiva of DED patients [64,65]. NF-κB activation pathways release pro-inflammatory cytokines (such as IL-1b, IL-2, IL-6, IL-8, IL-12, and TNF-α) and chemokines (MCP-1, IL-18, and CXCL 10), which can not only trigger an inflammatory response but also lead to goblet cell loss. Both pathways also induce the expression of adhesion molecules (ICAM-1, VCAM-1, and MMPs) that activate T-cell migration and result in corneal-barrier disruption and differentiation of CD4+T cells to T-helper cells [66]. The extent and effects of chronic inflammation combined with the dysfunction and loss of conjunctival goblet cells decrease the mucin levels present in human tears [50]. Also, the interrelationship between TNF-Alpha, NF-κB, and MMP-9 is well documented [67,68]. Numerous studies have demonstrated that the regulation of MMPs is a tightly controlled process, starting from the gene expression levels of IL-1β, NF-κB, and TNF-α, to the activation of zymogens and the internal inhibition mechanisms involving tissue inhibitors of MMPs [52,69,70,71].

In our clinical study, we presumably targeted NF-κB signaling. As shown in previous studies, helenalin and its derivatives abrogate NF-κB signaling by suppressing the DNA binding activity of NF-κB p65; this blockage is due to an inhibition of I-κB [15,16,17,18,19]. Additionally, it has also been demonstrated that helenalin suppresses CD4 cells via the mitochondrial pathway of apoptosis and by inducing G2/M cell cycle arrest [6,8,15,18,64]. By blocking NF-κB activation, helenalin can reduce the production of pro-inflammatory cytokines, chemokines, and other inflammatory mediators.

Our decision to focus primarily on MMP-9 as a marker for targeted NF-κB signaling, rather than explicitly measuring IL-1beta, IL-6, and TNF-α, was influenced by MMP-9′s established utility as a marker of inflammation in superficial eye diseases [52,72,73]. Its clinical relevance and ease of measurement, along with its significant role as an indicator of inflammatory disease activity, were key factors in our choice. Furthermore, the interrelationship between NF-κB, IL-1, IL-6, TNF-Alpha, and MMP-9 is well documented [67,68]. This provided us with a practical and clinically viable method to assess inflammatory activity.

This helenalin-based studied formulation has demonstrated clinical efficacy in treating DED. The underlying mechanism appears to be the regulation of inflammation evidenced by a statistically significant reduction of the MMP-9 positivity rate and the normalization of CIC, which results in a reduction of DED symptoms and clinical signs.

The main limitations of this study include a relatively small sample size, a single-center study, and a lack of stratified randomization. The short-term nature of the clinical follow-up, especially in the efficacy study, restricts our ability to assess the long-term effects of the helenalin-based formulation. On the other hand, the rapid and multi-targeted efficacy of the studied formulation aligns with previous outcomes of similar studies that blocked NF-κB/TNF-α pathways [65,74,75]. It is important to recognize that the rapid improvement observed in this study may not be representative of the standard response across all DED therapies. Therefore, additional studies are required to explain the cellular and molecular mechanism underlying the TNF-α signaling blockade by helenalin and the improved clinical outcomes in DED.

Meanwhile, different studies using ophthalmic solutions with active ingredients that block NF-κB/TNF-α pathways, like the presumed mechanism of helenalin in our formulation, have shown promising results in the therapy of DED. The ophthalmic solution of Tanfanercept (HBM9036), an anti-TNF-α monoclonal antibody, improves corneal staining scores, Schirmer’s scores, and TBUT in DED patients [76]. Also, HL036, a molecularly engineered TNF Receptor 1 fragment, improves corneal staining, reduces ocular discomfort, suppresses lacrimal inflammation, decreases corneal inflammation, and improves goblet cell counts by suppressing IFN-γ, IL-21, and IL-6 in a dry eye-induced C57BL/6 mice model [74]. Additionally, the topical anti-TNF-α-agent Licaminlimab, a single-chain antibody fragment that binds to and neutralizes the activity of human TNFα, improves ocular discomfort scores in patients with severe DED [77].

## 4. Materials and Methods

This clinical investigation engaged the participation of both healthy subjects’ eyes (pertaining to the safety and tolerability clinical study) and patients with mild-to-moderate DED (pertaining to the efficacy clinical study). DED was defined as patients presenting with an OSDI score of ≥13 along with at least one homeostasis marker, as defined by the DEWS-II diagnostic approach [78]. Patients were categorized as having mild-to-moderate DED, corresponding to Grades I and II of the Tear Film and Ocular Surface Society (TFOS) DEWS I severity classification [79]. To be included in the study, patients had to test positive for the presence of MMP-9 with an InflammaDry© (Quidel Eye Health, San Diego, CA, USA) test result exceeding 40 ng/mL. The trial, which was undertaken from January to May 2023, adhered to the ethical principles encapsulated in the Declaration of Helsinki and took place at ASG Clinical Retinal Research, an ophthalmological research center situated in Zapopan, Mexico. Before patient enrollment, the study received approval from the internal review board and the CRMQ Ethics and Research Committee, according to the local guidelines of the regulatory authorities for the conduction of clinical trials (COFEPRIS) (ID: CRMQ-OFT-001-2022), in full alignment with the International Conference on Harmonization on Good Clinical Practices. Informed written consent was obtained from all participants and their accompanying witnesses, after ensuring a comprehensive understanding of the study’s nature and potential adverse events.

### 4.1. Studied Formulation

The ophthalmic formulation under scrutiny was prepared in congruence with the principles of Good Manufacturing Practice and the Mexican Pharmacopoeia (FEUM) 13^th^ edition [39]. This sterile, translucent ophthalmic suspension, ensconced within a 15 mL container, was crafted to incorporate helenalin (0.008% to 0.015%) and dehydroalanine tiglate (5% to 7%), deriving their origins from a 1% extract of *Arnica montana*, which is included in the Mexican Herbal Pharmacopoeia [38] (chemical analysis is included in the Supplementary Material). A Drug to Extract Ratio (DER) falling within the gamut of 1:5 to 1:7 was achieved. Moreover, this formulation incorporated HA, presenting a concentration of 0.4%. The formulation’s pH was set at 7.4, complemented by a viscosity of 20 cPs. Noteworthy additions included ethylene diamine tetra-acetic acid (0.0005 g), benzalkonium chloride as a preservative (0.0002 g), hydroxypropylmethylcellulose (0.01 g), monobasic sodium phosphate (0.0003 g), dibasic sodium phosphate (0.0012 g), sodium chloride (0.009 g), sodium hydroxide (0.0001 g), and Grade 2 purified water (0.9647 g). This formulation adhered to Mexican regulations. The full list of compounds can be seen in Table 7.

### 4.2. Evaluation of Tolerability and Safety in Healthy Volunteers (Phase I Clinical Study)

**Figure 5:**
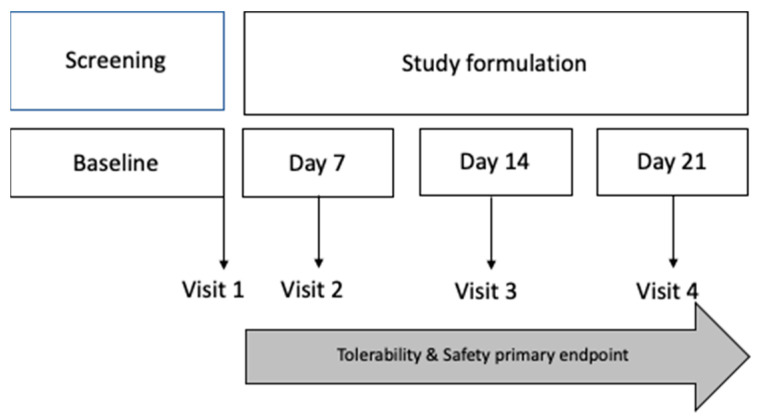
Tolerability and safety evaluation design. The safety and tolerability evaluation included the collection and summary of adverse events. Primary tolerability and safety analysis took place at visit 2 (day 7), visit 3 (day 14), and visit 4 (day 21).

A phase I clinical study was designed to assess the safety and tolerability of this ophthalmic formulation. Healthy individuals ranging from 18 to 65 years of age were selected to participate in this phase I study. Good health was defined as the absence of any medical or systemic surgical history. An Ocular Surface Disease Index (OSDI) < 13 [80,81], a non-invasive film tear breakup time (NIBUT) >10 s [82], absent corneal and conjunctival staining, and a negative result on the InflammaDry^®^ MMP-9 test were part of the inclusion criteria in this phase I [73]. Additionally, candidates were scrutinized for any ocular pathologic conditions, which, if present, led to exclusion from this study. To be eligible for this study, patients needed to be free of lubricant use in both eyes for at least six months before their enrollment. None of the patients enrolled in the phase I clinical study had undergone ocular surgery in the previous 6 months. Key exclusion criteria included systemic diseases associated with DED, a history of recurrent ocular inflammation, ocular-lid trauma, active ocular-lid infection, use of systemic trigger-dry eye drugs, any other ophthalmic solutions (such as antibiotics or pressure-lowering medications), and corneal abnormalities that could interfere with the study evaluation tests (such as ocular staining or allergy to fluorescein sodium or lissamine green dyes). Participants’ demographic and baseline clinical data were systematically compiled 5 days before the onset of the studied formulation application. After enrollment, participants were instructed to regularly apply one drop of the formulation three times a day to the study eye for 3 weeks. This was performed according to local regulations [83]. The choice of the eye for the study was randomized and determined by a coin toss. Follow-up was conducted the week after the application period, gauging any potential adverse events in alignment with tolerability and safety guidelines endorsed by FEUM [38] and Comisión para la Protección contra Riesgos Sanitarios-COFEPRIS (Figure 5). Compliance was also monitored; any value below 90% was tantamount to non-compliance and thereby excluded from statistical analysis.

Follow-up ocular evaluations included the measurement of BCVA adhering to the ETDRS protocol as well as the determination of IOP, according to the preferred practice pattern for comprehensive adult eye and vision examination [84]. Additionally, the quantification of corneal endothelial cell density (cECD) was performed through the utilization of specular microscopy (Perseus endothelial microscope from Costruzione Strumenti Oftalmici, Firenze, Italy). Additionally, slit lamp examinations were conducted at every visit, including examinations under white light and examinations with ocular surface staining using a cobalt blue filter and 1.0 mg fluorescein sodium ophthalmic strips (FluoroTouch^®^, Madhu Instruments, New Delhi, India). Furthermore, a slit lamp examination with lissamine green 1.5 mg dye-impregnated strips (GreenTouch^®^, Madhu Instruments, New Delhi, India) in conjunction with a red-free filter was performed. Ocular Adverse Events (AEs) were reported concurring with NOM-220-SSA1-2016, which contains the Mexican regulatory guidelines for the instillation and handling of commercial and research drugs, herbal medicines, medical devices, and their potential adverse events (including ophthalmic products) [83]. To reduce interobserver discrepancies, a single certified technician assessed safety and efficacy.

### 4.3. Evaluation of Efficacy in Patients with Mild-to-Moderate DED (Phase II Clinical Study)

We designed a phase II clinical study to evaluate the efficacy of the ophthalmic formulation. This was a prospective, randomized, double-blinded, crossover, interventional study comparing the studied formulation with commercially available lubricant eye drops. The inclusion criteria for this study were mild-to-moderate DED, based on OSDI score [80,81], a NIBUT < 10 s, corneal and conjunctival staining, the presence of visual strain, and a daily screen interaction of less than 8 h [78,80,85]. Patients required a positive test result in the InflammaDry© test, as confirmation of elevated levels of MMP-9 (positive test result in levels > 40 ng/mL) [73]. After meeting these criteria, participants were randomly assigned into one of two efficacy study groups, and they were subsequently assigned to either receive the trial ophthalmic formulation (Group 1) or a commercially available lubricant eye drop containing HA 0.04% (Group 2) (Linzaug^®^, Laboratorios Opko, Zapopan, México) as Control. Both eye drops were administered according to pre-specified protocols and frequencies (one drop three times a day in both eyes).

We aimed to ensure patient compliance and maintain the therapeutic effect while minimizing the irritation of the ocular surface associated with preservatives that we used at a frequency of three times a day, as recommended by the preferred practice pattern^®^ from the American Academy of Ophthalmology [86].

To evaluate the formulation’s efficacy, an evaluation strategy was devised, incorporating an array of ophthalmic parameters, tested on day 7, day 14, and day 28. These tests included the assessment of BCVA, the probing of non-invasive film tear breakup time (NIBUT) (using a Schwind Sirius+^®^ Topographer, SCHWIND Eye-Tech-Solutions, Kleinostheim, Germany) and non-invasive average breakup time (NIAvg-BUT), the evaluation of meibomian gland changes (using the Schwind Sirius^®^ Meibomiography, SCHWIND Eye-Tech-Solutions, Kleinostheim, Germany), and the detection of MMP-9 using the InflammaDry^®^ MMP-9 Test (Quidel Eye Health, San Diego, CA, USA). Additional evaluations included CIC, Schirmer’s test 1, ocular surface staining, and the ocular irritability test (for techniques and technical details, consult Appendix A). To reduce interobserver discrepancies, a single certified technician performed the BCVA measurement [87]. Safety and efficacy assessments were performed by a single, blinded, clinical investigator at each visit [87].

**Figure 6:**
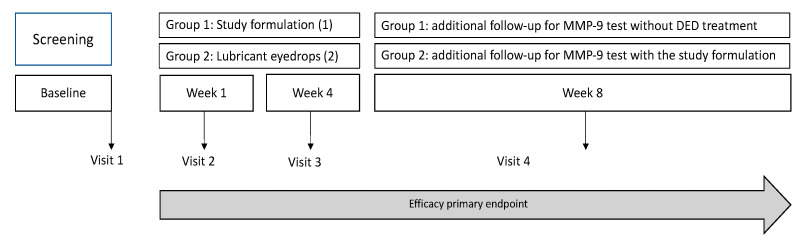
Efficacy evaluation design. Each subject underwent a baseline visit (visit 1) and was randomly assigned to 1 of 2 groups: 1 (studied formulation) and 2 (commercially available eye lubricant). Follow-up lasted 28 days for both efficacy study groups. An additional 1-month period of follow-up for MMP-9 testing was conducted in both groups.

For the crossover, an additional comprehensive 1-month follow-up period was implemented for MMP-9 testing exclusively, encompassing both study groups. Participants in the treatment group (Group 1) were directed to discontinue the application of the studied formulation, while those in the Control group (Group 2) initiated the administration of the studied formulation according to the established protocol, employing a crossover design without a washout period (Figure 6).

### 4.4. Data Analysis and Statistical Methods

The sample size determination employed a formula for comparing two independent means. Specifically, “nc” denotes the required sample size for the reference group (Group 2), while “ne” represents the sample size for the Study group (Group 1). The formula utilized is D = (Mc − Me), where “Mc” stands for the mean of the first group and “Me” signifies the mean of the second group. “S^2^ corresponds to the variance shared by both distributions, which is assumed to be equal. “Zß” denotes the standard normal function’s abscissa axis value, where the cumulative probability (1 − ß) is located. Assuming a mean difference in OSDI value of 22.4 OSDI units between treatment Group 1 and Group 2, with a standard deviation of 15 units (l), the study necessitated a minimum sample size of 19 subjects for each group to achieve a statistical power of 80% at a significance level of 5%, while considering a margin of superiority of 10 units. Accounting for a potential 20% loss to follow-up, it was determined that the final sample size for each group should consist of a minimum of 23 subjects. This calculation is summarized by the formula: “nc = ne = 2 × S^2^/D^2^ × (Zα/2 × Zß)^2^”.

Data analysis was conducted using IBM SPSS Statistics version 22.0 (IBM Corp, Armonk, NY, USA). Descriptive statistics were employed to characterize quantitative variables, including means and standard deviations, while qualitative variables were described in terms of frequencies and percentages. Given the small sample sizes (*n <* 30) and without making any assumptions about the normal distribution of the population, non-parametric tests were utilized for hypothesis testing. In the case of intragroup analysis involving related samples, the Wilcoxon rank test was utilized for assessing quantitative variables. For intergroup analysis comparing non-related samples, differences between groups were assessed using the Mann–Whitney U test. Significance was defined as a *p*-value less than 0.05 [88].

## 5. Conclusions

The helenalin-based formulation derived from *Arnica montana* and HA has demonstrated excellent tolerability and a good safety profile as a lubricant eye drop in individuals with healthy eyes. Additionally, it exhibits notable clinical efficacy by alleviating dry eye-related symptoms (as indicated by the OSDI score), enhancing tear film stability (measured by NIBUT, NIAvg-BUT, OSS, Schirmer’s test, and meibomiography), and exerting an anti-inflammatory effect (evidenced by the reduction in the MMP-9 positivity rate and the normalization of CIC) in patients afflicted with mild-to-moderate DED. These findings support the concept of an effective novel product for the management of DED. However, additional studies are required to explain the cellular and molecular mechanism underlying the improvement of clinical outcomes by the blockage of NF-κB/TNF-α pathways by helenalin.

Additionally, considering the favorable clinical outcomes and the acceptable safety profile exhibited by topical helenalin within this study, it is prudent to advocate for further comprehensive investigations on a larger cohort. It is worth emphasizing that addressing DED necessitates a multifaceted approach. Effective management requires the integration of a variety of strategies, including the use of lubricant eye drops, proper eyelid hygiene practices, and lifestyle modifications.

## 6. Patents

The senior author (AS) has developed 78 patent-protected inventions. 

## Figures and Tables

**Table 1:** Adverse events were reported in the healthy eyes of healthy subjects after the application of the studied formulation.

		Dry Eye Sensation	Burning	Discharge	Tearing	Blurred Vision
		*n* (%)	*n* (%)	*n* (%)	*n* (%)	*n* (%)
Frequency	Not presented	23 (95.83)	21 (87.5)	24 (100)	22 (91.66)	23 (95.83)
	Uncommon	1 (4.16)	1 (4.16)		1 (4.16)	1 (4.16)
	Occasionally		2 (8.33)		1 (4.16)	
	Most of time					
	All the time					
Severity	Mild	1 (4.16)	3 (12.5)			1 (4.16)
	Moderate				2 (8.33)	
	Severe					

**Table 2:** Clinical characteristics of eyes treated with the ophthalmic studied formulation.

				Baseline			Day 21		
Subject	Gender	Laterality	Age	BCVA	IOP	cECD	BCVA	IOP	cECD
			(Years)	(ETDRS-Letters)	(mmHg)	(µm)	(ETDRS-Letters)	(mmHg)	(µm)
1	F	OD	26	83	12	3189	83	11	3178
2	F	OD	27	84	11	3284	85	13	3190
3	M	OD	26	85	13	3182	84	17	3204
4	M	OD	26	83	13	3472	83	11	3488
5	M	OS	25	82	11	3323	84	14	3339
6	M	OS	25	84	14	3380	85	12	3382
7	F	OS	24	84	12	3452	84	12	3451
8	F	OS	26	83	12	3234	85	15	3230
9	F	OS	24	83	16	3085	85	13	3098
10	F	OS	25	83	11	3119	83	17	3127
11	M	OD	56	84	16	2874	84	15	2890
12	M	OD	35	82	17	2639	85	14	2620
13	M	OD	47	84	11	2572	85	15	2581
14	F	OD	36	85	18	2951	85	16	2947
15	F	OD	63	83	13	2383	85	14	2385
16	M	OS	56	83	15	2742	83	14	2746
17	F	OD	40	84	16	3183	84	14	3180
18	M	OS	38	84	14	2508	85	11	2519
19	M	OD	45	83	15	2823	84	13	2832
20	M	OS	28	82	12	2937	84	10	2926
21	F	OS	31	83	12	3166	83	16	3175
22	M	OD	39	82	16	2593	84	14	2603
23	F	OS	47	83	11	2496	84	15	2509
24	M	OS	59	83	15	2269	85	16	2276

OD; right eye, OS; left eye, BCVA; best corrected visual acuity, ETDRS; Early Treatment Diabetic Retinopathy Study, IOP; intraocular pressure, cECD; corneal endothelial cell density.

**Table 3:** Baseline characteristics of subjects and eyes of the phase II study.

	Group 1 (Studied Formulation)	Group 2 (Control/Commercially Available Eye Lubricant)
Age	53.1 ± 9.82	52.5 ± 13.15 ‡
Gender		‡
Male (*n*)	12	12
Female *(n)*	12	12
Hypertension *(n)*	1	2
Ocular findings:		
Pseudophakic *(n)*	5	3
Basal BCVA (ETDRS letters)	82.5 ± 4.2	82.2 ± 5.1 ‡
OSDI (score)	21.31 ± 3.21	21.74 ± 15 ‡
NIBUT (s)	8.39 ± 5.86	8.43 ± 4.82 ‡
NIAvg-BUT (s)	10.46 ± 4.19	9.85 ± 4.13 ‡
Schirmer’s test (mm)	15.83 ± 5.4	16.11 ± 5.1 ‡
MMP-9 Test positivity	(24/24) 100%	(24/24) 100% ‡
Impression cytology with abnormal characteristics	8/24 (33.3%)	7/24 (29.1%) ‡

BCVA: Best Corrected Visual Acuity, ETDRS: Early Treatment Diabetic Retinopathy Study, IOP: intraocular pressure, MMP-9: Matrix Metalloproteinase-9, NIBUT: non-invasive film tear breakup time, NIAvg-BUT: non-invasive average breakup time value, OSDI: Ocular Surface Disease Index, ‡: no statistically significant difference between groups using the Mann–Whitney U test (*p* > 0.05).

**Table 4:** Quantitative variables’ analysis in DED patients exposed to the studied formulation (Group 1) or commercially available lubricant eye drops (Group 2).

	Group 1		Group 2	
Variable/Visit	B	1-Month	B	1-Month
OSDI (score)	21.31 ± 3.21	10.58 ± 4.21 *	21.74 ± 15	11.23 ± 8.95 *
NIBUT (s)	8.39 ± 5.86	14.53 ± 4.53 *	8.43 ± 4.82	13.83 ± 5.69 *
NIAvg-BUT (s)	10.46 ± 4.19	14.24 ± 3.66 *	9.85 ± 4.13	11.73 ± 5.82
Schirmer’s test (mm)	15.83 ± 5.4	20.05 ± 4.37 *	16.11 ± 5.1	17.83 ± 6.28
MMP-9 elevated levels	24/24 (100%)	6/24 (25%) *	24/24 (100%)	21/24 (87.5%)
Impression cytology with abnormal characteristics	8/24 (33.3%)	0/0 (0.0%) *	7/24 (29.1%)	7/24 (29.1%)

B: Baseline, MMP-9: Matrix Metalloproteinase-9, NIBUT: non-invasive film tear breakup time, NIAvg-BUT: non-invasive average breakup time, OSDI: Ocular Surface Disease Index, *: statistically significant difference between baseline and 1-month using the Wilcoxon test (*p* < 0.05)

**Table 5:** Millipore membrane impression cytology (grading system by Nelson) for Group 1 vs. Group 2.

	Group 1		Group 2	
Number of Subjects *	Baseline	1-Month	Baseline	1-Month
1	1	0	1	1
2	2	0	3	3
3	3	0	2	2
4	2	0	1	2
5	2	0	2	2
6	3	0	3	3
7	2	0	2	2
8	2	0		

All eyes with ocular surface abnormalities in Group 1 showed an improvement, after a 1-month treatment period. None of the eyes in Group 2 showed an improvement. There was a significant statistical difference in Group 1′s impression cytology baseline vs. 1-month (*p*-value = 0.0078) and Group 1 vs. Group 2 (*p*-value = 0.0002). Group 2 did not show significant statistical differences (*p*-value = 0.9999 and 0.5052, respectively). The impression cytology scores after treatment with the studied formulation and control group were 2.21 vs. 0.00 for Group 1 and 2.00 vs. 2.00 in Group 2. 1: Studied formulation group (*n* = 8); 2: Control group, commercially available lubricant eye drops (*n* = 7); Millipore membrane impression cytology, Nelson gradation: normal or minimal changes, with intact goblet cells and predominantly normal epithelial cells (Grade 0); mild metaplasia, characterized by some cells and increase in non-goblet epithelial cells (Grade 1); moderate metaplasia, with significant loss of goblet cells and increased proliferation of non-goblet epithelial cells (Grade 2); and severe metaplasia, marked by the complete absence of goblet cells and a predominance of squamous epithelial cells. * Number of subjects with abnormal CIC at baseline.

**Table 6:** Ocular Surface Staining Score (OSS) and ocular surface staining with fluorescein and lissamine green in dry eye patients exposed to the studied formulation (Group 1) and Control group (Group 2).

	Group 1		Group 2	
Grade/Visit	Baseline	1-Month	Baseline	1-Month
0	2	18	1	3
I	8	6	11	9
II	9	0	6	8
III	3	0	6	4
IV	2	0	0	0
V	0	0	0	0

Quantitative variables’ analysis in DED patients exposed to studied formulation drops (mean ± SD). Grading of staining should be absent in non-DED eyes (Grade 0). This table shows the result of the fluorescein (F) and lissamine green (LG) staining, which compares the baseline and 1-month intakes of Group 1 and Group 2 study groups.

**Table 7:** Composition of studied formulation (per 100 g).

Components	Concentration (grams)
Ethylene Diamine Tetra-acetic Acid	0.05
Benzalkonium Chloride	0.02
Hydroxypropylmethylcellulose	1.00
Monobasic Sodium Phosphate	0.03
Dibasic Sodium Phosphate	0.12
Arnica Montana Extract	1.00
Sodium Chloride	0.90
Sodium Hydroxide	0.01
Sodium Hyaluronate	0.40
Injectable Grade Water	96.47

Note: Concentrations are presented in grams (g) per 100 g of formulation.

## Data Availability

Data presented in this study are available upon request from the corresponding author, arturo.santos@tec.mx.

## Appendix A

The objective evaluation involved a baseline ophthalmologic clinical examination, with subsequent assessments on day 7, day 14, and day 28. The evaluations encompassed various ophthalmic parameters, including:

- Best Corrected Visual Acuity (BCVA), according to ETDRS protocol.
- Non-invasive film tear breakup time (NIBUT) and non-invasive average breakup time (NIAvg-BUT) using the Schwind Sirius+ topographer (CSO SRL, Italy).
- Meibomiography with Schwind Sirius: Meiboscores were assigned based on Grades 0 to 3 for meibomian gland changes.
- MMP-9 evaluation with InflammaDry MMP-9 Test (right eye only): Sampling fleece dabbed on lower eyelid conjunctiva; test assembled as per manufacturer’s instructions.
- Conjunctival impression cytology (CIC) (left eye only): Millipore filter pressed onto palpebral conjunctiva, sample fixed with ethyl alcohol, and graded using Nelson system.

The following are the Nelson gradation system grades:

Grade 0: Normal or minimal changes, intact goblet cells, normal epithelial cells.

Grade 1: Mild metaplasia, altered cells, increased non-goblet epithelial cells.

Grade 2: Moderate metaplasia, reduced goblet cells, increased non-goblet epithelial cells.

Grade 3: Severe metaplasia, absence of goblet cells, prevalence of squamous epithelial cells.

- Schirmer test 1 (without anesthesia) for tear secretion volume (Eagle Vision, Inc., Memphis, TN, USA).

Ocular surface staining: F and LG staining using 1.5 mg strips with 1% reagent, assessed according to SICCA guidelines.

- Intraocular pressure (IOP) with Icare TA01i tonometer; fundus assessment with binocular indirect ophthalmoscope.
- Comprehensive safety assessment involved collecting and summarizing ocular and non-ocular AEs across evaluations.

All assessments occurred in a controlled environment (humidity: 40–50%, temperature: 23–25 °C), with compliance monitored through a patient care journal. Adherence < 90% led to exclusion. Withdrawal was allowed but alternative DED treatments were not. Changes from baseline to day 28 in various tests were considered efficacy endpoints. Diagnostic criteria included OSDI > 13, BUT < 10 s, Schirmer < 10 mm/5 min, and positive staining score.

The OSDI questionnaire (Spanish version) was used for subjective ophthalmic assessment.

A certified technician conducted BCVA measurement, while a blinded clinical investigator performed safety and efficacy assessments. A crossover design included a 4-week follow-up for MMP-9 testing, with Group 1 discontinuing the formulation and Group 2 initiating it without a washout period.
